# High dose dexamethasone treatment for Acute Respiratory Distress Syndrome secondary to COVID-19: a structured summary of a study protocol for a randomised controlled trial

**DOI:** 10.1186/s13063-020-04646-y

**Published:** 2020-08-26

**Authors:** Luis Patricio Maskin, Gabriel Leonardo Olarte, Fernando Palizas, Agostina E. Velo, María Fernanda Lurbet, Ignacio Bonelli, Natalio D. Baredes, Pablo Oscar Rodríguez

**Affiliations:** 1 Intensive Care Unit, CEMIC, Buenos Aires, Argentina; 2 Pulmonary Section, Internal Medicine Department, CEMIC, Buenos Aires, Argentina; 3 Intensive Care Unit, Sanatorio Sagrado Corazon, Buenos Aires, Argentina; 4 Intensive Care Unit, Clinica Bazterrica, Buenos Aires, Argentina

**Keywords:** COVID-19, Dexamethasone, Acute Respiratory Distress Syndrome, Randomised controlled trial, protocol, Steroids

## Abstract

**Objectives:**

The aim of this study is to explore the effectiveness and safety of high dose dexamethasone treatment for Acute Respiratory Distress Syndrome secondary to SARS-Cov-2 pneumonia.

**Trial design:**

Multicentre, randomized clinical trial, controlled, open label, parallel group, to evaluate the effectiveness and safety of high dose dexamethasone in adult patients with confirmed COVID-19, with Acute Respiratory Distress Syndrome.

**Participants:**

We will include patients with SARS-Cov-2 pneumonia who develop acute respiratory distress syndrome, in several intensive care units (ICU) in Buenos Aires, Argentina (CEMIC, Clinica Bazterrica, Sanatorio Sagrado Corazon)

Inclusion criteria:

- Men and women, age ≥ 18 years old.
- Confirmed diagnosis of SARS-CoV-2 infection, by RT-PCR.
- Diagnosis of Acute Respiratory Distress Syndrome (hypoxemic respiratory failure not explained by cardiac disease + PaO_2_/FiO_2_ ratio < 300 with a Positive End-Expiratory Pressure ≥ 5 cm H_2_O + bilateral pulmonary infiltrates)
- Length of mechanical ventilation of at least 72 hours
- Informed consent (next of kin / legal guardian)

Exclusion criteria:

- Pregnant or breast-feeding women.
- Terminal disease (advanced cancer; under palliative care; cardiovascular, respiratory, or renal disease with a life expectancy less ≤ 1 year).
- Therapeutic limitation (advance directives or do not resuscitate order)
- Severe immunosuppression (HIV infection, long-term use of immunosuppressive agents, active cancer).
- Patients under chronic treatment with glucocorticoids for other diseases (≥ 8 mg prednisone, or equivalent)
- Participation in another randomized clinical trial.

**Intervention and comparator:**

Eligible patients will be randomized to receive standard ICU patient care (group 1) or standard ICU patient care plus high dose dexamethasone (group 2).

- Group 1: dexamethasone up to 6 mg/24 hours for up to 10 days + ventilatory, hemodynamic, nutritional, and antimicrobial support according to international guidelines.
- Group 2: dexamethasone 16 mg/24 hours for 5 days followed by dexamethasone 8 mg/24 hours for 5 days + ventilatory, hemodynamic, nutritional, and antimicrobial support according to international guidelines.

**Main outcome:**

The main result is ventilator-free days at 28 days (Days without ventilator support in the first 28 days following randomization). Secondary outcomes are 28-days and 90-days mortality, frequency of nosocomial infections in the first 28 days after randomization, Sequential Organ Failure Assessment (SOFA) score variation and prone position in the first 10-days, viral shedding 28-days after randomization, and delirium and muscle weakness at ICU discharge.

**Randomisation:**

Treatment will be assigned according to site stratified randomization by permuted random blocks sequence 1:1 generated with a table in R language concealed in a randomization tool in REDCap (Research Electronic Data CAPture) platform.

**Blinding (masking):**

This is an open trial, so no masking of treatment assignment will be used.

**Numbers to be randomised (sample size):**

Assuming a 3 days difference in ventilator-free days between treatment groups, with a mean of 9 days, and a standard deviation of 9 days; the necessary sample size would be 284 subjects (142 per group), with a power of 80% and a two-tailed alpha error of 0.05.

**Trial Status:**

The protocol with code 1264, version 3.0 on date: May 13, 2020 is approved by the local Ethics Committee. The trial is in the recruitment phase. Recruitment began May 22, 2020 and is anticipated to be complete by the end of December 2021.

**Trial registration:**

The trial was registered under the title “Dexamethasone for COVID-19 Related ARDS: a Multicenter, Randomized Clinical Trial” with ClinicalTrials number NCT04395105, https://clinicaltrials.gov/ct2/show/NCT04395105, registered on 20 May 2020.

**Full protocol:**

The full protocol is attached as an additional file, accessible from the Trials website (Additional file 1). In the interest in expediting dissemination of this material, the familiar formatting has been eliminated; this Letter serves as a summary of the key elements of the full protocol.

## Supplementary information

**Additional file 1.** Study protocol.

## Data Availability

The data will be available from the author on reasonable request. Please contact the corresponding author Dr Pablo O. Rodriguez (prodriguez@cemic.edu.ar).

