# Supplementary material for: High dose dexamethasone treatment for Acute Respiratory Distress Syndrome secondary to COVID-19: a structured summary of a study protocol for a randomised controlled trial
Source: Trials. 2020 Aug 26;21:743. doi: 10.1186/s13063-020-04646-y (PMC7447582; doi:10.1186/s13063-020-04646-y)
Supplement: Supplementary file 1 — Additional file 1. Study protocol. [file 13063_2020_4646_MOESM1_ESM.docx]

ADDITIONAL FILE 1: STUDY PROTOCOL

**Protocol code:** DEXA-ARDS-COVID19 version 3.0, 13/MAY/2020

**Title:** HIGH DOSE DEXAMETHASONE TREATMENT FOR THE ACUTE RESPIRATORY DISTRESS SYNDROME SECONDARY TO COVID-19

**Sponsor:** Centro de Educación Medica e Investigaciones Clínicas Norberto Quirno (CEMIC)

**Principal Investigators:**

Dr. L Patricio Maskin

Pulmonary and Critical Care

Hospital Universitario CEMIC

Av Coronel Diaz 2423

1425 Ciudad de Buenos Aires, Argentina

email: [pmaskin@cemic.edu.ar](mailto:pmaskin@cemic.edu.ar)

Dr Pablo Oscar Rodríguez

Pulmonary and Critical Care

Hospital Universitario CEMIC

Av Coronel Diaz 2423

1425 Ciudad de Buenos Aires, Argentina

email: [prodriguez@cemic.edu.ar](mailto:prodriguez@cemic.edu.ar)

Clinicaltrials: NCT04395105

Centers of execution: Hospital Universitario CEMIC, Clinica Bazterrica, Sanatorio Sagrado Corazon, Ciudad de Buenos Aires, Argentina.

Study drug: dexamethasone

INTRODUCTION

The acute respiratory distress syndrome (ARDS) represents a spectrum of acute respiratory failure with diffuse, bilateral lung injury, severe hypoxemia and increased respiratory system stiffness, caused by non-cardiogenic pulmonary edema. Failure may be initiated by pulmonary or extrapulmonary insults. It poses high morbidity and mortality (1–3), and the main treatment is ventilatory support applied by mechanical ventilation. Pathophysiologically, ARDS can be divided in two phases, exudative (acute) and fibrotic (late)(4). The acute phase is characterized by both endothelial and epithelial injury, interstitial and alveolar edema with accumulation of neutrophils, macrophages, and red blood cells in the alveoli, and the activation of inflammation and coagulation cascades. Their complex interactions end in a neutrophilic alveolitis, with barrier disruption and interstitial edema, which is expressed as alterations in gas exchange and pulmonary mechanics (5,6). There is no specific pharmacologic therapy for ARDS; all treatments are aim at its underlying causes.

Emerge of coronavirus SARS-CoV-2 pneumonia (COVID-19) in Wuhan, China in 2019, and it posterior spread to the world, has exposed an overloaded health system in many regions. COVID-19 has been associated to variable clinical presentation and prognosis as preliminary reports are published (7–9). Available data indicates that most part of the cases were pausi- or asymptomatic, as mild forms of the disease are expressed. Report for New York (9) described the initial evolution of 5700 patients, 373 of whom (14.2%) needed intensive care admission, and 320 (12.2%) required mechanical ventilation. However, mortality under mechanical ventilation was 88.1%, and ARDS was probably the cause to initiate ventilatory support. In the Lombardia case-series (7), 1150/1591 critically ill patients needed mechanical ventilation, probably due to ARDS. These data indicate that the severe ill patients with SARS-CoV-2 infections usually require mechanical ventilation for long-periods, and their prognosis is worse.

Several treatment options had been tried since the initial description of COVID-19, such as antiviral, immunomodulators drugs, and immunotherapy. No treatment has shown a clear benefit at the present time. Lopinavir/ritonavir, a protease inhibitor for HIV, was evaluated in a randomized open trial, with no placebo, in in-patients with severe pneumonia (10). No significant differences were seen in the primary outcome (time to recover), though, it decreased the length of stay in intensive care. Remdesivir, an antiviral drug designed to treat Ebola, has shown in-vitro activity against SARS-CoV-2, and decreased the hospital length of stay without affecting mortality. Chloroquine has been used in SARS 2012 epidemic, due to activity against SARS-CoV. Preliminary reports suggested that hydroxychloroquine shorten viral shedding time in mild disease, especially in combination with azithromycin (11). Convalescent plasma has been evaluated in small series and one unconcluded randomized clinical trial with encouraging results (12,13). On the other hand, immunomodulatory therapies, such as steroids, interferon or antibodies against interleukine-6, had been proposed to treat the cytokine storm associated to coronavirus severe disease.

Systemic steroids have been the immunosuppressant drugs most used against COVID-19, in opposition to the initial recommendations (14). The inflammatory response in the acute phase and the frequent use of steroids in pulmonary interstitial diseases are the probably reasons for its use. However, there is concern in the medical community about the generalized use of immunosupressors (above all steroids) in COVID-19 infections. The efficacy of systemic steroids has been highly controversial in viral pneumonias prior to this pandemic. It is known that, in general, these drugs reduce lung inflammation but also inhibit the immune response and clearance of the pathogen. The fear of a negative effect on viral replication, and the increase of hospital infection spread, are the main reasons for avoidance of corticosteroid prescription. These concerns are based in low quality observational and heterogeneous studies of influenza pneumonia, as indicated in a Cochrane review (15). However, some authors sustained that the inflammatory response is the main factor related to patient prognosis, with no relationship to viral shedding (16), and they recommended in favor of the utilization of steroids in severe cases.

The utility of steroids in the treatment of ARDS has been controversial for years. A recent statement of the SCCM (Society of Critical Care Medicine) and the ESICM (European Society of Intensive Care Medicine) suggest the utilization of methylprednisolone for 14 days in patients with early ARDS (17). This recommendation was based on the positive results in mortality and duration of mechanical ventilation of 4 clinical studies. Recently, Villar and cols published the results of a multicentre study performed in Spain, in which moderate to severe ARDS patients were randomized to dexamethasone (20 mg qd for 5 days followed by 10 mg qd for 5 days) against placebo (18). A decrease in hospital mortality (21% vs. 36%) and shorten in ventilators days (4.8 days difference) were reported with the use of dexamethasone. Main cause of ARDS in the study was pneumonia. Additional advantages of dexamethasone, in opposition to other steroids, are the administration period (once a day) and the lack of mineralocorticoid effect.

The Surviving Sepsis Campaign COVID-19 panel supported a weak recommendation to use steroids in the sickest patients with ARDS due to SARS-CoV-2 (19). Cochrane could not issue a report on the benefits of steroids, due to the absence of evidence in patients with COVID-19, and asked for time until the results of ongoing trials (19). A recommendation against the general use of steroids in patients with COVID-19 was initially issued by the NIH (National Institute of Health), the WHO (World Health Organization), the CDC (Center for Disease Control), and the IDSA (Infectious Disease Society of America), due to the absence of clear evidence (20,21).

Recently, the RECOVERY trial provides evidence that treatment with dexamethasone at a dose of 6 mg once daily for up to 10 days reduces 28-day mortality in patients with Covid-19 who are receiving respiratory support (22). This British study compared multiple treatments for COVID-19 in a randomized, open label trial, with a complex design, in patients with different severities. This sub study randomized 6425 patients (ratio 1:2) to received dexamethasone 6 mg/day for ten days against placebo. A decreased in 28-days mortality (21.6% vs. 24.6%) was seen as main outcome (age-adjusted rate ratio 0.83; IC 95% 0.74 a 0.92; P<0.001). The benefit was greater in the more severe patients who needed oxygen (n=3883; 21.5% vs. 25.0%, rate ratio 0.80 IC 95% CI 0.70 a 0.92; p=0.002) or mechanical ventilation (n=1007; 29.0% vs. 40.7%, rate ratio 0.65 IC 95% 0.51 a 0.82; p<0.001). These results were presented before the publication, and several institutions, such as the NHS and the NIH, changed their recommendation in favour of the use of dexamethasone (6 mg qd for 10 days).

The preliminary results of the RECOVERY trial made us change our original design for the usual care. In the beginning, this study sought to compare dexamethasone (higher doses than RECOVERY trial) versus usual care, without steroids. This approach is unfeasible nowadays, thus we modified our initial design to allow low dose dexamethasone for 10 days as usual care treatment. However, the dose proposed by the British study might not be enough to suddenly reverse the inflammatory changes in patients with ARDS.

In summary, there is evidence that a dysregulated inflammatory response in patients with COVID-19 pneumonia may cause ARDS with unfavorable prognosis. Many of them will require mechanical ventilation for long time, overcrowding the regional health system. The treatments of the severe COVID-19 pneumonia in unknown and randomized trials are needed to test their efficacy. The steroids, as unspecific anti-inflammatory agents, may shorten the respiratory failure and improve the prognosis. Low-lose dexamethasone reduced the risk of death in patients with oxygen needs, or mechanical ventilation. The aim of this study is to explore the effectiveness and safety of high-dose dexamethasone, against usual care, in the treatment of SARS-CoV-2 pneumonia, in patients with ARDS.

MATERIALS AND METHODS

TRIAL DESIGN

Multicenter randomized clinical trial, controlled, open, parallel group, to evaluate the effectiveness and safety of high dose dexamethasone in adult patients with confirmed COVID-19, with Acute Respiratory Distress Syndrome.

MAIN OUTCOMES

The main result is ventilator-free days at 28 days (Days without ventilator support in the first 28 days following randomization).

Secondary outcomes measures

• 28-days mortality (Dead rate within 28 days of randomization)

• 90-days mortality (Dead rate within 28 days of randomization)

• Frequency of nosocomial infections (number of ventilator-associated pneumonia, blood stream infection or candidemia in the first 28 days following randomization)

• Viral shedding (Frequency of positive PCR on nasopharyngeal swab 28 days after randomization)

• Serum C - reactive protein variation (10 days after randomization)

• SOFA variation (over the first 10 days after randomization)

• Use of prone position (Cumulative hours spent on prone position at 10 days after randomization)

• Delirium (Frequency of delirium at ICU discharge)

• Muscle weakness (mMRC score at ICU discharge)

PARTICIPANTS

We will include patients with SARS-Cov-2 pneumonia who develop acute respiratory distress syndrome, in several intensive care units (ICU) in Buenos Aires, Argentina.

Inclusion criteria:

- Men and women, with age ≥ 18 years old.
- Diagnosed SARS-CoV-2 infection, by PCR.
- Diagnosis of Acute Respiratory Distress Syndrome (hypoxemic respiratory failure not explained by cardiac disease + PaO2/FiO2 ratio < 300 with a Positive End-Expiratory Pressure ≥ 5 cm H2O + bilateral pulmonary infiltrates)(23)
- Need of mechanical ventilation in the last 72 hours
- Informed consent (next of kin/ legal guardian)

Exclusion criteria:

- Pregnant or breast-feeding women.
- Terminal disease (advance cancer; under palliative care; cardiovascular, respiratory, or renal disease with a life expectancy less ≤ 1 year).
- Therapeutic limitation (advance directives or do not resuscitate order)
- Severe immunosuppression (HIV infection, long-term use of immunosuppressive agents, active cancer).
- Patients under chronic treatment with glucocorticoids for other diseases (dose ≥ 8 mg prednisone, or equivalent)
- Participation in another study.

INTERVENTION AND COMPARATOR

Eligible patients will be randomized to receive standard ICU patient care (group 1) or standard ICU patient care plus high dose dexamethasone (group 2).

- Group 1: dexamethasone up to 6 mg/24 hours for 10 days + ventilatory, hemodynamic, nutritional, and antimicrobial support according to international guidelines.
- Group 2: dexamethasone 16 mg/24 hours for 5 days and dexamethasone 8 mg/24 hours for 5 days + ventilatory, hemodynamic, nutritional, and antimicrobial support according to international guidelines.

Standard ICU patient care will not be regulated by the protocol. However, it is suggest treating the patients according to the international guidelines for ARDS (24), antibiotics and hemodynamic support for COVID-19 infection (19,25). The following standards are recommended:

- Sepsis: prompt infection source identification and adequate antibiotic treatment
- Hemodynamic support: individualized resuscitation with fluids and vasopressors to achieve adequate perfusion (mean arterial pressure target: 65-75 mm Hg)
- Protective mechanical ventilation
  - Tidal volume: 6 ml/kg (ideal body weight)
  - Plateau pressure < 30 cm H_2_O, Driving pressure < 15 cm H_2_O
  - Respiratory rate to achieve PaCO_2_ 35-45 mm Hg
  - Minimal FiO_2_ to achieve SaO_2_ 88-94%
  - PEEP titration, according to ARDSnet trial (26), or Express maximal recruitment (27)
- Dexamethasone use: no more than 6 mg qd for ten days

Weaning protocol

The weaning protocol is defined by each site. We recommend evaluating the patients in a daily basis to perform a spontaneous breathing trial (SBT), based on the ARDSnet protocol, with a FiO_2_ <= 0.5. The following are the minimal pre-requisites to perform an SBT:

- PaO_2_/FiO_2_ > 200 con PEEP <= 10 cm H_2_O (>12 hours)
- Hemodynamic stability
- Minimal sedation (RASS> -1)
- Cough during aspiration maneuvers

The trial will be performed in pressure support ventilation (pressure support 8-10 cm H_2_0, and ZEEP, with a FiO_2_ <= 50%), if possible. If the test is passes successfully, the attending physician will decide, or not, to extubate the patient. This test can be done daily.

POSSIBLE CONFOUNDERS

The following variables will be evaluated as possible confounders:

- Age and gender
- Ventilatory mode, and parameters of mechanical ventilation
- Oxygenation variables (pH, PaO_2_, PaCO_2_)
- Severity scores (APACHE II (28), SOFA (29))
- Shock
- Co morbidities (Charlson score (30))

RANDOMISATION AND BLINDING (MASKING)

Treatment will be assigned according to site stratified randomization by permuted random blocks sequence 1:1 generated with a table in R language. The randomization table is concealed in the randomization tool of the REDCap (Research Electronic Data CAPture) platform.

This is an open trial, so no masking of treatment assignment will be used.

SAMPLE SIZE

Assuming a 3 days difference in ventilator-free days between treatment groups, with a mean of 9 days, and a standard deviation of 9 days; the necessary sample size would be 284 subjects (142 per group), with a power of 80% and a two-tailed alpha error of 0.05.

STATISTICAL ANALYSIS:

All tests will be on an intention-to-treat basis. Initially, a descriptive analysis will be performed. The different variables will be compared according to the treatment with usual statistical test according to data distribution (normality of data will be evaluated by Shapiro-Wilk test).

Main outcome variable analysis (ventilator free-days) will be compared by Wilcoxon test. A competing risk model will be done for the time to first successful extubation, adjusting for APACHE II score and ARDS severity. Two competing events (successful weaning and dead under mechanical ventilation) will be evaluated; sub-hazard ratio will be calculated by the Fine and Gray method.

Survival at 28 days will be evaluated by Cox regression analysis, with assigned treatment, APACHE II and ARDS severity as predictors. Change in different daily parameters (vital sign, SOFA score, chemistry, sedation, parameters of mechanical ventilation, among others) will be compared by a mixed-effect regression model (fixed and random), using the treatment as fixed variable and the patient code and the day of data acquisition as random. The same procedure will be performed for safety measures, using glucose levels and insulin requirements. Number of infections in the first 28 days will be analyzed with a Poisson model, with treatment, APACHE II score, and ARDS severity as predictors. A quasi-Poisson model, or a negative binomial, will be done in case of over-dispersion. In this case the results will be expressed to the best fit model, according to Akaike Information Criterion (AIC).

The analysis will be carried out with the statistical package R: A language and environment for statistical computing (R Core Team, R Foundation for Statistical Computing, Vienna, Austria. URL <http://www.R-project.org/>).

TRIAL REGISTRATION AND CURRENT STATUS

The trial was registered under the title “Dexamethasone for COVID-19 Related ARDS: a Multicenter, Randomized Clinical Trial” with ClinicalTrials number NCT04395105, registered on 20 May 2020. The protocol with code DEXA-ARDS-COVID19, version 3.0 on date: 13/MAY/2020 is already approved by the Local Research Ethics Committee of CEMIC. The trial is already in the recruitment phase.

ETHICS APPROVAL AND CONSENT TO PARTICIPATE

The protocol was evaluated by the Local Research Ethics Committee of CEMIC (Buenos Aires, Argentina), on May 13, 2020 with the reference code 1264. It was approved on May 13, 2020. The study was also approved by the SATI (Sociedad Argentina de Terapia Intensiva) Ethics Committee.

Patient’s next of kin will be asked for informed consent. This will be reflected in the medical record before included in the clinical trial. A copy of the informed consent will be attached in the medical record.

REFERENCES

1. Bertolini G, Lewandowski K, Bion J, Romand J-A, Villar J, Thorsteinsson A, et al. Epidemiology and outcome of acute lung injury in European intensive care units. Intensive Care Med. 2004 Jan 1;30(1):51–61.

2. Rubenfeld GD, Caldwell E, Peabody E, Weaver J, Martin DP, Ph D, et al. Incidence and Outcomes of Acute Lung Injury. N Engl J Med. 2005;353:1685–93.

3. Needham DM, Dowdy DW, Mendez-Tellez PA, Herridge MS, Pronovost PJ. Studying outcomes of intensive care unit survivors: measuring exposures and outcomes. Intensive Care Med. 2005 Sep 21;31(9):1153–60.

4. Blondonnet R, Constantin J, Sapin V, Jabaudon M. A Pathophysiologic Approach to Biomarkers in Acute Respiratory Distress Syndrome. Dis Markers. 2016;2016:1–20.

5. Matthay MA, Ware LB, Zimmerman GA. Review series The acute respiratory distress syndrome. J Clin Invest. 2012;122(8):2731–40.

6. Spadaro S, Park M, Turrini C, Tunstall T, Thwaites R, Mauri T, et al. Biomarkers for Acute Respiratory Distress syndrome and prospects for personalised medicine. J Inflamm. 2019 Dec 15;16(1):1.

7. Grasselli G, Zangrillo A, Zanella A, Antonelli M, Cabrini L, Castelli A, et al. Baseline Characteristics and Outcomes of 1591 Patients Infected with SARS-CoV-2 Admitted to ICUs of the Lombardy Region, Italy. JAMA - J Am Med Assoc. 2020;323(16):1574–81.

8. Guan W, Ni Z, Hu Y, Liang W, Ou C, He J, et al. Clinical characteristics of coronavirus disease 2019 in China. N Engl J Med. 2020;382(18):1708–20.

9. Richardson S, Hirsch JS, Narasimhan M, Crawford JM, McGinn T, Davidson KW, et al. Presenting Characteristics, Comorbidities, and Outcomes among 5700 Patients Hospitalized with COVID-19 in the New York City Area. JAMA - J Am Med Assoc. 2020;323(20):2052–9.

10. Cao B, Wang Y, Wen D, Liu W, Wang J, Fan G, et al. A trial of lopinavir-ritonavir in adults hospitalized with severe covid-19. N Engl J Med. 2020;382(19):1787–99.

11. Gautret P, Lagier J-C, Parola P, Hoang VT, Meddeb L, Mailhe M, et al. Hydroxychloroquine and azithromycin as a treatment of COVID-19: results of an open-label non-randomized clinical trial. Int J Antimicrob Agents. 2020;(xxxx):105949.

12. Shen C, Wang Z, Zhao F, et al. Treatment of 5 Critically Ill Patients With COVID-19 With Convalescent Plasma. J Am Med Assoc. 2020;(29):1–8.

13. Duan K, Liu B, Li C, Zhang H, Yu T, Qu J, et al. Effectiveness of convalescent plasma therapy in severe COVID-19 patients. Proc Natl Acad Sci. 2020;1–7.

14. Tobaiqy M, Qashqary M, Al-Dahery S, Mujallad A, Hershan AA, Kamal MA, et al. Therapeutic Management of COVID-19 Patients: A systematic review. medRxiv. 2020;2020.04.02.20051029.

15. Lansbury LE, Rodrigo C, Leonardi-Bee J, Nguyen-Van-Tam J, Shen Lim W. Corticosteroids as Adjunctive Therapy in the Treatment of Influenza: An Updated Cochrane Systematic Review and Meta-analysis. Crit Care Med. 2020;48(2):e98–106.

16. Villar J, Confalonieri M, Pastores SM, Meduri GU. Rationale for Prolonged Corticosteroid Treatment in the Acute Respiratory Distress Syndrome Caused by Coronavirus Disease 2019. Crit Care Explor. 2020 Apr;2(4):e0111.

17. Annane D, Pastores SM, Rochwerg B, Arlt W, Balk RA, Beishuizen A, et al. Guidelines for the diagnosis and management of critical illness ‑ related corticosteroid insufficiency ( CIRCI ) in critically ill patients ( Part I ): Society of Critical Care Medicine ( SCCM ) and European Society of Intensive Care Medicine ( ESICM ) 20. Intensive Care Med. 2017;

18. Villar J, Ferrando C, Martínez D, Ambrós A, Muñoz T, Soler JA, et al. Dexamethasone treatment for the acute respiratory distress syndrome: a multicentre, randomised controlled trial. Lancet Respir Med. 2020;8(3):267–76.

19. Alhazzani W, Møller MH, Arabi YM, Loeb M, Gong MN, Fan E, et al. Surviving Sepsis Campaign : guidelines on the management of critically ill adults with Coronavirus Disease 2019 ( COVID ‑ 19 ). Intensive Care Medicine. Springer Berlin Heidelberg; 2020.

20. Cheng VC, Edwards KM, Gandhi R, Muller WJ. Infectious Diseases Society of America Guidelines on the Treatment and Management of Patients with COVID-19. 2020;

21. World Health Organization. WHO Clinical management of severe acute respiratory infection (SARI) when COVID-19 disease is suspected. Who. 2020;(March):12.

22. Article O. Dexamethasone in Hospitalized Patients with Covid-19 — Preliminary Report. N Engl J Med. 2020 Jul 17;NEJMoa2021436.

23. Definition TB. Acute Respiratory Distress Syndrome. JAMA. 2012 Jun 20;307(23).

24. Care I, Papazian L, Aubron C, Brochard L, Chiche JD, Combes A, et al. Formal guidelines : management of acute respiratory distress syndrome. Ann Intensive Care. 2019;

25. Cecconi M, De Backer D, Antonelli M, Beale R, Bakker J, Hofer C, et al. Consensus on circulatory shock and hemodynamic monitoring. Task force of the European Society of Intensive Care Medicine. Intensive Care Med. 2014 Dec 13;40(12):1795–815.

26. England TN. Ventilation with Lower Tidal Volumes as Compared with Traditional Tidal Volumes for Acute Lung Injury and the Acute Respiratory Distress Syndrome. N Engl J Med. 2000 May 4;342(18):1301–8.

27. Mercat A, Richard J-CM, Vielle B, Jaber S, Osman D, Diehl J-L, et al. Positive End-Expiratory Pressure Setting in Adults With Acute Lung Injury and Acute Respiratory Distress Syndrome. JAMA. 2008 Feb 13;299(6):646.

28. Knaus W, Draper E, Wagner D, Zimmerman J. APACHE II: a severity of disease classification system. Crit Care Med. 1985; 13(10):818-29

29. Vincent J, Moreno R, Takala J, Willatts S, Mendon A De, Reinhart CK, et al. The SOFA ( Sepsis . related Organ Failure Assessment ) score to describe organ dysfunction / failure On behalf of the Working Group on Sepsis . Related Problems of the European Society of Intensive Care Medicine ( see contributors to the project in the ap. Intensive Care Med. 1996;22:707–10.

30. Charlson M, Pompei P, Ales K, MacKenzie C. A new method of classifying prognostic comorbidity in longitudinal studies: developement and validation. J Chronic Dis. 1987;40:373–83.
